# Notes on the Use of Bacteriological Methods of Diagnosis in Private Practice.—IV

**Published:** 1898-10-29

**Authors:** J. O. Symes

**Affiliations:** Bacteriologist to the Royal Infirmary, Bristol.


### MEDICAL PROGRESS AND HOSPITAL CLINICS.

### NOTES ON THE USE OF BACTERIOLOGICAL METHODS OF DIAGNOSIS IN PRIVATE PRACTICE.—IV.

By J. O. Symes, M.D.Lond., Bacteriologist to the Royal Infirmary, Bristol.

(*Concluded from page* 8.)

**Urinary Diseases.**—A bacteriological examination of the urine may be required for the purposes of diagnosis in tuberculous disease of the kidneys, ureters, and bladder, in suppurative nephritis, pyelitis, or cystitis in bacteruria, in enteric fever, and other general infections. Urine in the healthy bladder contains no micro-organisms, but these abound at the meatus urinarius and anterior two inches of the urethra. To obtain a pure specimen, either of the two following methods may be adopted: (1) Cleanse the meatus and surrounding parts with a solution of perchloride of mercury, 1 in 1,000. Allow a little water to be passed, and then collect the remainder in a sterilised vessel. (2) Cleanse the meatus with antiseptic solution, and pass a catheter that has been previously boiled and lubricated with glycerine. The receiving vessel should be immediately plugged. The specimen is now allowed to stand until a deposit forms, the excess of fluid is poured off, and films are prepared from the deposit. As there is but little albuminous material in urine, bacilli may not be firmly fixed to the coverslip, so that after staining the cover-glasses should not be washed under the tap, but the stain, with the aid of a few drops of water, should be sucked off with blotting-paper. If tuberculous disease be suspected the films are stained by Ziehl Neelsen's method, whilst for other micro-organisms Löffler's methylene blue is employed. Although it is occasionally possible to detect bacilli in the sediment obtained by allowing the specimen to stand, it will more often be found that it is necessary to employ the centrifuge. With the aid of the centrifuge tubercle bacilli may easily be demonstrated if present, and the accompanying tissue débris may help to indicate the portion of the urinary tract involved by the disease. Accidental contamination by the smegma bacillus, which has similar staining reactions to the tubercle bacillus, need not be feared if the proper precautions have been taken in securing the specimen.

In typhoid fever the bacillus typhosus is to be detected in the urine after the second or third weeks, but the specimen requires to be centrifugalised, and plate cultures made. Staphylo and strepto-cocci may be demonstrated in cases of suppuration in the urinary tract, generally without centrifugalisation. In bacteruria, organisms such as B. coli are frequently so abundant as to give a distinctly cloudy appearance to the urine. Films from urine are prepared by taking a portion of the sediment in the sterilised platinum loop, placing the drop between two coverslips, drawing them apart, and fixing by passing through the flame.

**Pulmonary Diseases.**—A bacteriological examination of the sputum is sometimes required in diseases other than phthisis—in pneumonia, putrid bronchitis, bronchiectasis, actinomycosis, gangrene of the lung, and influenza. To demonstrate the presence of the pneumococcus, a small quantity of the viscid gelatiniform sputum is spread between coverslips, fixed, and stained with methylene blue. The lanceolate cocci are found in pairs, having a capsule around. The fact that they are not decolorised by Gram's stain serves to distinguish them from the pneumobacillus. In many cases of lobar pneumonia the sputum will be found to contain the pneumococcus in almost pure culture and in immense numbers. In broncho-pneumonia, on the other hand, the stained films show chiefly strepto- or staphylococci, bacillus coli, or other organisms. Films prepared from the sputum of putrid bronchitis may exhibit a large number of micro-organisms, amongst which may be recognised the bacillus of Lumniezer. Still more numerous are the organisms in chronic bronchitis and bronchiectasis, cocci, moulds, sarcinæ, leptothrix, and bacilli being met with in large numbers. The bacillus of influenza may be detected by means of cover-slip preparations of the sputum of persons suffering from the disease. These bacilli are not easily stained with methylene blue, but good preparations can be made by staining for ten minutes in a solution of carbol fuchsin diluted with water.

**Post-mortem Examinations.**—Cultures or preparations made for purposes of diagnosis may be made after death either from localised lesions or, in the case of general infections, from the blood or organs.

It is important that the examination should be made as soon after death as possible, for after the lapse of 24 or 36 hours the tissues are frequently found to be invaded by the bacillus coli or other allied organisms, the growth of which may completely mask the existence of the organism which was the determining cause of death. This invasion of the tissues by bacillus coli is, we think, particularly frequent in cases of disease of the abdominal viscera. In localised suppurations, such as meningitis, pericarditis, or deep-seated abscesses, the method of examination is as follows: The abscess cavity should be opened with a knife that has previously been sterilised. A drop of pus is taken in the loop of a sterilised needle, and spread as thinly as possible over a series of cover-glasses, and fixed by passing through the flame. The films are subsequently stained and examined under the microscope.

When the cover-glass preparations are made the needle is again sterilised in the flame and a second drop of the pus inoculated upon the surface of culture tubes.

In taking cultures from the solid organs the most useful will generally be found to be the spleen and the heart, unless, indeed, the nature of the disease should point to the more probable implication of some other viscus. The method most commonly employed consists in searing the surface of the organs with a heated knife or cautery, cutting through the burned area with a sterilised knife and then thrusting a sterilised platinum needle into the opening so made. An alternative method consists in sterilising the surface of the organ by soaking for half an hour in 1 in 1,000 perchloride of mercury, drying, cutting through the capsule with a sterilised knife, and then taking scrapings with the platinum needle or other available instrument. In either case smear preparations on coverslips are made, and then with fresh material tubes are inoculated. Such cultures taken after death are of especial service in confirming diagnosis in doubtful cases of pneumonia, pyæmia, septicæmia, enteric fever, diphtheria, &c.

